# Common Polymorphisms in Human Langerin Change Specificity for Glycan Ligands*[Fn FN3]^♦^

**DOI:** 10.1074/jbc.M113.528000

**Published:** 2013-11-11

**Authors:** Hadar Feinberg, Thomas J. W. Rowntree, Shawn L. W. Tan, Kurt Drickamer, William I. Weis, Maureen E. Taylor

**Affiliations:** From the § Department of Life Sciences, Imperial College, London SW7 2AZ, United Kingdom and; the ‡ Departments of Structural Biology and Molecular and Cellular Physiology, Stanford University School of Medicine, Stanford, California 94306

**Keywords:** Carbohydrate-binding Protein, Crystal Structure, Genetic Polymorphism, Glycobiology, Lectin, CD207, Sulfated Glycans

## Abstract

Langerin, a C-type lectin on Langerhans cells, mediates carbohydrate-dependent uptake of pathogens in the first step of antigen presentation to the adaptive immune system. Langerin binds a diverse range of carbohydrates including high mannose structures, fucosylated blood group antigens, and glycans with terminal 6-sulfated galactose. Mutagenesis and quantitative binding assays indicate that salt bridges between the sulfate group and two lysine residues compensate for the nonoptimal binding of galactose at the primary Ca^2+^ site. A commonly occurring single nucleotide polymorphism (SNP) in human langerin results in change of one of these lysine residues, Lys-313, to isoleucine. Glycan array screening reveals that this amino acid change abolishes binding to oligosaccharides with terminal 6SO_4_-Gal and enhances binding to oligosaccharides with terminal GlcNAc residues. Structural analysis shows that enhanced binding to GlcNAc may result from Ile-313 packing against the *N*-acetyl group. The K313I polymorphism is tightly linked to another SNP that results in the change N288D, which reduces affinity for glycan ligands by destabilizing the Ca^2+^-binding site. Langerin with Asp-288 and Ile-313 shows no binding to 6SO_4_-Gal-terminated glycans and increased binding to GlcNAc-terminated structures, but overall decreased binding to glycans. Altered langerin function in individuals with the linked N288D and K313I polymorphisms may affect susceptibility to infection by microorganisms.

## Teaser

**Background:** Langerin is a receptor in the immune system that binds sugars on pathogens.

**Results:** The effects of genetic variations that cause changes in the sugar-binding site of human langerin have been investigated.

**Conclusion:** Two common, linked, variations in the gene for human langerin change sugar specificity of langerin.

**Significance:** Individuals with variant forms of langerin may have altered susceptibility to pathogenic microorganisms.

## Introduction

Langerin (CD207), a C-type lectin of Langerhans cells, can act as a pathogen receptor by binding to surface glycoconjugates of microorganisms (1–3). Pathogens bound by langerin include *Mycobacterium leprae* (4), viruses such as HIV, herpes simplex virus 2, and measles virus (5–7), and fungi including *Malassezia furfur* and *Candida* species (8, 9). Langerin mediates internalization of glycoconjugates into Birbeck granules, which are subdomains of the endosomal compartment unique to Langerhans cells, as the first step in antigen processing and presentation, the main function of Langerhans cells (1, 7, 10, 11). Langerin protects against HIV infection by internalizing the virus to Birbeck granules for degradation (5, 12).

The extracellular portion of langerin contains a C-type carbohydrate recognition domain (CRD)^2^ that binds sugars and a neck region that mediates formation of trimers stabilized by a coiled-coil of α-helices (13). Langerin binds to a diverse set of glycan ligands including high mannose structures, β-glucans, and fucosylated blood group antigens (8, 9, 13–15). As in other C-type lectins, the CRD of langerin contains a primary Ca^2+^-dependent sugar-binding site where Ca^2+^ forms direct coordination bonds with two vicinal hydroxyl groups of a monosaccharide (16–18). Crystal structures of langerin-ligand complexes show that specificity for high mannose oligosaccharides, β-glucans, and the blood group B trisaccharide results from binding of a single mannose, glucose, or fucose residue to the Ca^2+^ at the primary sugar-binding site and a small number of favorable contacts with other portions of the oligosaccharide ligands beyond the monosaccharide in the primary binding site (18).

**FIGURE 1.**
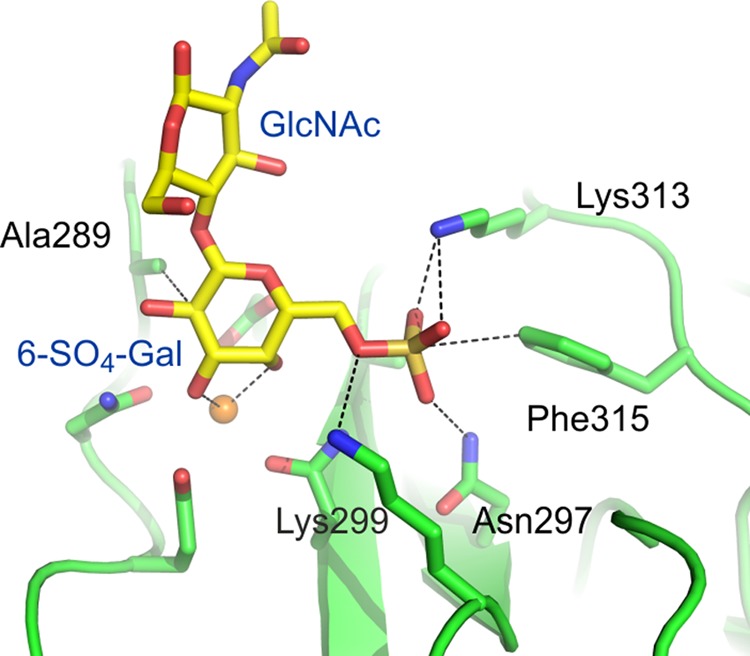
**Mechanism of 6SO_4_-Gal binding by langerin.** Langerin CRD bound to 6SO_4_-Galβ1-4GlcNAc (18). The protein is shown in *green*, Ca^2+^ is in *orange*, oxygen atoms are *red*, nitrogen atoms are *blue*, and the sulfur atom is *gold*.

Uniquely for a C-type lectin with the characteristic mannose-type binding motif Glu-Pro-Asn at the primary Ca^2+^- and sugar-binding site (19, 20), langerin can also bind galactose-type ligands. Langerin binds glycans with terminal 6-sulfated galactose but not those with terminal 3-sulfated galactose or nonsulfated galactosides (9, 14, 15). The equatorial 3- and axial 4-OH groups of galactose ligate to the Ca^2+^ at the primary sugar-binding site with salt bridges formed between two lysine residues and the sulfate group (see Fig. 1) (18). The nonoptimal Ca^2+^ ligation of galactose at the primary sugar-binding site appears to be compensated by the charge-charge interactions between the sulfate group and the two lysine residues. Glycans with terminal 6SO_4_-Gal are not typical markers of pathogens. However, an endogenous ligand, keratan sulfate, containing repeating 6SO_4_-Galβ1-4GlcNAc units is bound by langerin, suggesting a possible role for 6SO_4_-Gal recognition by langerin in cell adhesion (9). Recently, other glycosaminoglycans, especially heparin, have been shown to interact with langerin, but this binding is Ca^2+^-independent and appears to be due to charge-charge interactions with part of the trimeric neck region, not due to binding to the CRD (21).

The human langerin gene contains many single nucleotide polymorphisms (SNPs) including some that cause amino acid changes in the CRD and affect sugar binding activity (22). The studies described here characterize the effects of a polymorphism that changes Lys-313, one of the amino acid residues critical for binding 6SO_4_-Gal-terminating glycans, to isoleucine, which is linked to another SNP that changes Asn-288 to Asp. A combination of mutagenesis, glycan array screening, and structural studies shows that the K313I change alters the specificity of langerin for glycans in an unexpected way, not only abolishing binding to 6SO_4_-Gal-containing ligands, but also causing enhanced binding to glycans with terminal GlcNAc.

## EXPERIMENTAL PROCEDURES

### 

#### 

##### Protein Expression and Purification

Amino acid changes corresponding to the polymorphisms in the CRD of human langerin and other mutations were introduced into the human langerin cDNA using synthetic oligonucleotides. Expression and purification of wild type and mutant forms of the langerin CRD and of a trimeric fragment of the extracellular domain containing the CRD plus part of the neck region (truncated langerin) were produced using bacterial expression systems as described previously (13, 15).

##### Analysis of Ligand Binding

Specificity of wild type and mutant langerin for 6SO_4_-Gal and mannose was determined using a solid-phase binding assay with truncated langerin immobilized to polystyrene wells (13). Fluorescein-labeled truncated langerin, prepared as described previously (15), was used to probe version 5.1 of the glycan array following the standard procedure of Core H of the Consortium for Functional Glycomics.

##### Analysis of Langerin SNPs in Genomic DNA

Human genomic DNA samples were obtained from the European Collection of Cell Cultures (Salisbury, UK). Part of the langerin gene containing exons 5 and 6 was amplified from human genomic DNA samples by PCR using forward primer 5′-gctccaagtgtggccgcacctctg-3′ and reverse primer 5′-gagctcaaagagtgagcttgggag-3′. Amplified DNA was sequenced on an ABI Prism 310 genetic analyzer. For analysis of individual alleles from genomic DNA samples heterozygous for langerin SNPs, PCR products were cloned into plasmid CR II TOPO using the Topo cloning kit (Invitrogen), and clones were sequenced.

##### Crystallization and Data Collection

The crystals of langerin CRD, langerin-I313 CRD, and langerin-D288/I313 CRD complexed with carbohydrate ligands were obtained by hanging-drop vapor diffusion, using a mixture of 1.5–2 μl of protein-carbohydrate complex and 0.75–1 μl of reservoir solution in a drop. Crystals were grown at 22 °C from a protein solution comprising 2.3–4.0 mg/ml langerin, 2.5 mm CaCl_2_, 10–100 mm Tris (pH 8.0), 25 mm NaCl with excess of sugar, as follows. GlcNAc (Sigma) or α-Me-GlcNAc (Sigma) was added at 50 mm to form complexes with langerin-I313 CRD or langerin-D288/I313 CRD. GlcNAc (50 mm) or α-Me-GlcNAc (60 mm) was added to form the wild type langerin CRD complexes. α-Me-Man (Sigma) at 50 mm was used to form the complex with langerin-D288/I313 CRD. Trisaccharides GlcNAcβ1-3Galβ1-4Glcβ-CH_2_CH_2_N_3_ and GlcNAcβ1-3Galβ1-4GlcNAcβ-CH_2_CH_2_N_3_ (compounds TR54 and TR55 from Core D of the Consortium for Functional Glycomics) were added to the protein solution at a final concentration of 15 mm. The reservoir solution contained 0.1 m HEPES (pH 7.0), 0.1–0.2 m MgCl_2_, and 20–35% polyethylene glycol 3350. All crystals, except for the complex of langerin CRD with trisaccharide TR54, were transferred to perfluoropolyether (PFO-XR75; Lancaster Synthesis) before being frozen in liquid nitrogen for data collection. The crystal of langerin CRD with trisaccharide TR54 was frozen directly from the crystallization drop. Diffraction data for the complexes of langerin CRD and langerin-I313 CRD with α-Me-GlcNAc or GlcNAc were measured at 100 K on beamline 5.0.3 at the Advanced Light Source. Diffraction data for the complexes of langerin CRD, langerin-I313 CRD, and langerin-D288/I313 CRD with trisaccharide TR54 or TR55 and for the langerin-D288/I313 CRD with α-Me-GlcNAc and α-Me-Man were measured at 100 K on beamline 11-1 at the Stanford Synchrotron Radiation Lightsource. Data were processed with MOSFLM or XSD and scaled with SCALA.

##### Structure Determination

The crystals belonged to space group P4_2_ with unit cell parameters similar to those published for other langerin CRD complexes, allowing structure solution directly by rigid body refinement (18). The model from Protein Data Bank entry 3P5G, without the blood group B trisaccharide or water molecules, was used as the starting model for the refinement of the complex of langerin. For all the data sets, the same reflections were marked as the test set as in the data for langerin with blood group B trisaccharide. The crystallographic asymmetric unit contains four copies of the CRD. Model building and refinement were performed with Coot and PHENIX (23, 24). Refinement included individual positional and isotropic temperature factor refinement for all atoms, except in some structures where the Ca^2+^ was refined with an anisotropic temperature factor. All models start at residue 196, 197, or 198 and end at residue 324 or 325. The sugar directly bound to the principal Ca^2+^ is visible in all copies. The second and third sugars of the trisaccharides were added to the model when they were clearly visible in the electron density maps.

## RESULTS

### 

#### 

##### A Naturally Occurring Polymorphism in Langerin That Affects Binding to 6SO_4_-Gal

Several single nucleotide polymorphisms in the human langerin gene that cause nonsynonymous amino acid changes have been reported in the National Center for Biotechnology Information (NCBI) SNP database (www.ncbi.nlm.nih.gov/snp). The effects of three of these amino acid changes in the CRD on langerin function were previously investigated: A278V (SNP database cluster identification rs741326), N288D (SNP database cluster identification rs13383830), and A300P (SNP database cluster identification rs2080391) (22). Subsequent to the earlier studies, SNP rs57302492, which results in the change of lysine at position 313 to isoleucine, has been identified. The crystal structure of the CRD of langerin in complex with 6SO_4_-Galβ1-4GlcNAc shows that Lys-313 is one of two lysine residues that form salt bridges with the SO_4_ group of 6SO_4_-Gal, making it likely that the K313I change would impair binding to 6SO_4_-Gal-containing ligands (18).

To place the impact of this novel polymorphism in context, mutagenesis studies on residues in the langerin 6SO_4_-Gal-binding site were conducted to establish the importance of the interactions observed in the crystal structure. The interactions with the 3- and 4-OH groups of the galactose moiety, through the principal Ca^2+^ ion, are common to the C-type CRDs (16). Interactions unique to the langerin-binding site for 6SO_4_-Gal include the salt bridges between the SO_4_ group and Lys-313 and Lys-299 and packing of Ala-289 against the galactose ring (Fig. 1) (18). This packing results from tipping of galactose relative to mannose bound at the same site, reflecting the different geometry of the 3- and 4-OH groups of galactose as compared with mannose.

**FIGURE 2.**
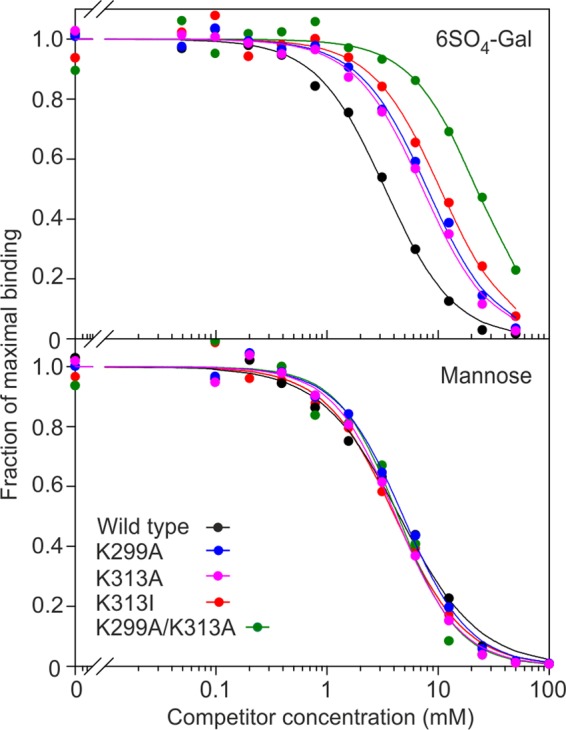
**Effect of Lys-299 and Lys-313 mutations on binding of 6SO_4_-Gal to langerin.** Inhibition of binding of the reporter ligand ^125^I-labeled mannose-BSA to langerin immobilized in polystyrene wells by mannose or 6SO_4_-Gal is demonstrated. Results are representative of two experiments performed in duplicate.

The contributions of Lys-313, Lys-299, and Ala-289 to binding of 6SO_4_-Gal were assessed by quantifying the impact of mutations at these positions in a trimeric fragment of the extracellular domain of langerin, designated truncated langerin (15). Comparing the inhibition constants for mannose and 6SO_4_-Gal indicates that wild type langerin has similar affinities for these two sugars with the *K_I_* for 6SO_4_-Gal about 1.4 times that of mannose (Table 1 and Fig. 2). Mutation of either Lys-313 or Lys-299 to alanine significantly reduces affinity for 6SO_4_-Gal without affecting mannose binding, and mutation of both lysine residues to alanine reduces binding of 6SO_4_-Gal to a weak level similar to that seen for galactose (Table 1 and Fig. 2). This residual level of binding reflects the fact that free galactose monosaccharide can bind weakly at the principal Ca^2+^ of langerin through interaction with the 2-OH group and the anomeric hydroxyl group (13). Such binding is an artifact because the anomeric hydroxyl would not be available in an oligosaccharide ligand. The loss of affinity for 6SO_4_-Gal in the double mutant supports the hypothesis that the salt bridges formed between the sulfate group of 6SO_4_-Gal and the two lysine side chains are necessary to compensate for the unfavorable orientation of galactose binding. Mutation of Ala-289 to serine resulted in a small reduction in affinity for 6SO_4_-Gal relative to mannose with *K_I_*_,Man_/*K_I_*_,Gal 6-SO4_ of 0.87, indicating that interaction of Ala-289 with the pyranose ring of galactose may also make a small contribution to 6SO_4_-Gal binding.

**TABLE 1:** **Binding of 6SO_4_-Gal to langerin** Relative inhibition constants for mannose, 6SO_4_-Gal, and galactose were determined in binding competition assays in which the reporter ligand ^125^I-labeled mannose-bovine serum albumin was bound to langerin immobilized in polystyrene wells. *K_I_* values represent mean ± S.D. of two independent assays performed in duplicate.

Mutant langerin	*K_I_*_,Man_	*K_I_*_,6SO4-Gal_	*K_I_*_,Gal_	*K_I_*_,Man_/*K_I_*_, 6SO4-Gal_
	*mm*	*mm*	*mm*	
Wild type	4.4 ± 0.1	3.1 ± 0.4	27.6 ± 0.1	1.44
K299A	4.8 ± 0.1	7.8 ± 0.4		0.62
K313A	3.8 ± 0.6	6.7 ± 0.9		0.56
K299A/K313A	4.1 ± 0.4	21.8 ± 0.8	16.3 ± 0.7	0.19
K313I	4.2 ± 0.1	10.2 ± 0.4		0.41

Mutation of Lys-313 to isoleucine, mimicking the naturally occurring variation, has a similar effect to the K313A mutation, with a 4-fold decrease in the *K_I_* for 6SO_4_-Gal as compared with wild type (Table 1 and Fig. 2). Thus, it seems likely that langerin function would be altered in individuals with the Ile-313 form of langerin.

##### Linkage of the K313I Polymorphism to Another Polymorphism, N288D

**FIGURE 3.**
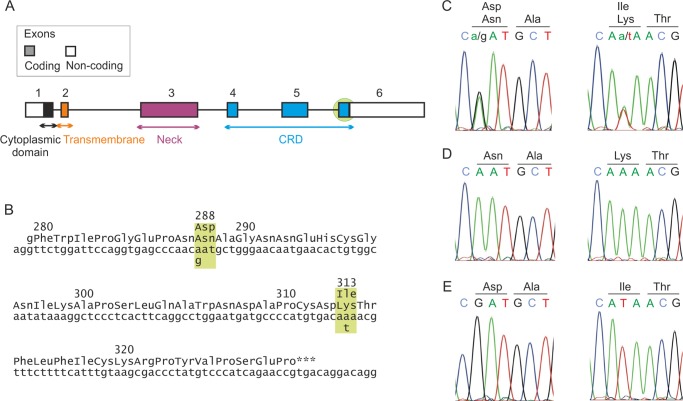
**Genomic analysis of langerin polymorphisms.** *A*, exon structure of the langerin gene. The *pale green circle* indicates the region of the gene that was sequenced. *B*, sequence of the coding region of exon 6 showing the positions of N288D and K313I polymorphisms. *C*, sequence of parts of exon 6 for an individual heterozygous for N288D and K313I. *D*, sequence of allele 1 from the heterozygous individual showing Asn-288 and Lys-313. *E*, sequence of allele 2 from the heterozygous individual showing Asp-288 and Ile-313.

Genomic DNA samples from several different populations were analyzed to assess the frequency of the K313I polymorphism. DNA corresponding to exon 6 of the langerin gene, which encodes amino acids 280 to the C terminus, was amplified by PCR and sequenced directly. Several individuals heterozygous for the Ile-313 variant were identified but no homozygotes (Fig. 3 and Table 2). Exon 6 of the langerin gene also contains two other SNPs that affect langerin function, causing the changes N288D and A300P, so it was possible to look for these changes at the same time. None of the individuals had proline at position 300, confirming that this is a rare change. However, all of the individuals heterozygous for the amino acid at position 313 were also heterozygous for the N288D polymorphism. No individuals homozygous for Asp at position 288 were found. To determine whether the codons specifying Ile-313 and Asp-288 were present on the same chromosome in each of the heterozygous individuals, the PCR products obtained were cloned, and several independent clones were sequenced. Only two types of clones with respect to the polymorphisms at positions 288 and 313 were found, one coding for Asn-288 and Lys-313, and another coding for Asp-288 and Ile-313. These results indicate that the two polymorphisms are linked, with the common allele encoding Asn-288 and Lys-313 and the variant form encoding Asp-288 and Ile-313. The frequency of the minor allele was highest in the African samples.

**TABLE 2:** **Frequencies of K313I and N288D polymorphisms determined by sequencing of exon 6 of the langerin gene from human genomic DNA samples**

Population	Number of samples	Position 313			Position 288		
		AAA/AAA (Lys/Lys)	AAA/ATA (Lys/Ile)	ATA/ATA (Ile/Ile)	AAT/AAT (Asn/Asn)	AAT/GAT (Asn/Asp)	GAT/GAT (Asp/Asp)
Black African	20	15	5	0	15	5	0
Japanese	9	8	1	0	8	1	0
Italian	13	12	1	0	12	1	0
French	7	7	0	0	7	0	0
South American	2	2	0	0	2	0	0
Indian							
Thai	2	2	0	0	2	0	0
Oriental	2	2	0	0	2	0	0

Frequency data are available for these two polymorphisms in the SNP database (www.ncbi.nlm.nih.gov/snp). In all populations studied, the allele frequencies for the K313I polymorphism are identical to those for the N288D polymorphism. Data from the 1000 Genomes Phase 1 study are consistent with the PCR data showing that the K313I polymorphism and the N288D polymorphism are linked. The minor allele frequency for each of the two polymorphisms is 10.7%. In each case, the minor allele was seen 233 times in the population of 1092 people or 2184 chromosomes. For each polymorphism, 12 of the 1092 individuals are homozygous for the minor allele. In every case where an individual is homozygous for Ile at position 313, they are also homozygous for Asp at position 288, and individuals heterozygous for Ile at position 313 are also heterozygous for Asp at position 288. In the 1000 Genomes study, the frequency of the allele with Asp-288 and Ile-313 is highest in African populations (19%), intermediate in the Asian populations (12%), and lowest in European and American populations (6%).

##### Changes in Langerin Glycan Specificity Resulting from the K313I Polymorphism

Residue 288 is near the principal Ca^2+^ site to which the sugar binds, and the previous analysis shows that the N288D change reduces sugar binding affinity (22). Thus, langerin with both Asp-288 and Ile-313 was predicted to have reduced affinity for all ligands as well as impaired 6SO_4_-Gal binding activity. Glycan array screening was used to determine the effects of the K313I and N288D amino acid changes on specificity of langerin for glycan ligands. A version of the truncated, trimeric langerin mimicking the naturally occurring minor allele with Asn-288 mutated to aspartate and Lys-313 changed to isoleucine (langerin-D288/I313) was created and compared with the form with only the K313I change (langerin-I313) and wild type langerin. Previous glycan array analysis of langerin was carried out using version 3.2 of the glycan array of the Consortium for Functional Glycomics (15). Here, version 5.0 of the glycan array, which is an expanded array containing 611 glycans, was screened.

**FIGURE 4.**
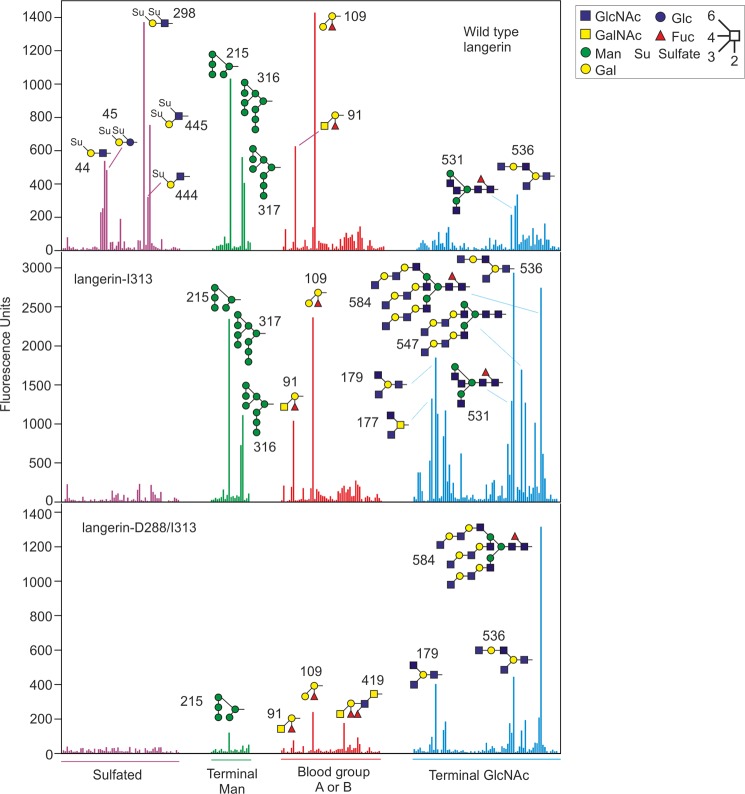
**Glycan array analysis of langerin variants.** Ligands giving the highest signals are identified. Glycans are grouped based on similar structures. *Purple* indicates sulfated glycans, *green* indicates structures with terminal mannose residues, *red* denotes glycans containing the blood group A or B trisaccharides, and GlcNAc-terminated glycans are indicated in *blue*. The complete list of glycans included in the figure is provided in supplemental Table 1.

In agreement with previous glycan array screening, wild type langerin is seen to bind to high mannose structures (glycans 215, 316, and 317), some blood group A- or B-containing glycans (glycans 91 and 109), and glycans with terminal 6SO_4_-Gal, including glycans 298, 444, 445, 44, and 45, but not to other types of sulfated glycans (Fig. 4) (15). Glycan array analysis of langerin with isoleucine at position 313 shows that binding to 6SO_4_-Gal-terminated glycans is abolished by this amino acid change because none of the ligands with terminal 6SO_4_-Gal that bind to wild type langerin are bound by langerin-I313 (Fig. 4). This result is consistent with the large reduction in affinity for 6SO_4_-Gal caused by the loss of lysine at position 313. In contrast, binding to high mannose structures and fucosylated blood group antigens is unaffected (Fig. 4) because binding of langerin-I313 to blood group A- or B-containing glycans (glycans 91 and 109) and high mannose structures (glycans 215, 316, and 317) is similar to that of wild type langerin. Interestingly, langerin-I313 shows enhanced binding of many GlcNAc-terminated glycans, for example glycans 536, 584, 547, 177, 179, and 531, as compared with wild type langerin, which binds only weakly to these glycans.

Screening of the glycan array with langerin-D288/I313 shows that this form of langerin has similar specificity to langerin-I313, with no binding to glycans with terminal 6SO_4_-Gal residues and enhanced binding to GlcNAc-terminated glycans (Fig. 4). However, this form of langerin shows much weaker binding of the fucosylated blood group antigens and high mannose structures that are bound by wild type langerin and langerin-I313. The results shown for langerin-D288/I313 are from a screen performed in the presence of 20 mm CaCl_2_. Under the usual screening conditions of 2 mm CaCl_2_, the signals are lower, although the pattern of ligands bound is similar, with most significant binding to the GlcNAc-terminated ligands (data not shown). These results are consistent with previous experiments showing that the change of Asn-288 to aspartate results in a decrease in the affinity of the carbohydrate recognition domain for Ca^2+^ and thus a decrease in sugar binding affinity (22). Comparison of the glycan array results for langerin-D288/I313 and langerin-I313 indicates that the change of lysine to isoleucine at position 313 results in altered specificity for glycan ligands, from 6SO_4_-Gal- to GlcNAc-terminated glycans, whereas the N288D change decreases the affinity for glycans.

##### Structural Basis for Enhanced Binding of GlcNAc

To determine the molecular basis for the enhanced binding of GlcNAc-terminated glycans caused by the K313I change, crystal structures of the carbohydrate recognition domains of langerin-I313, langerin-D288/I313, and wild type langerin in complex with GlcNAc, α-Me-GlcNAc, and two GlcNAc-terminated trisaccharides (GlcNAcβ1-3Galβ1-4Glcβ-CH_2_CH_2_N_3_ (TR54) and GlcNAcβ1-3Galβ1-4GlcNAcβ-CH_2_CH_2_N_3_ (TR55)), representing the terminal portions of poly *N*-acetyllactosamine chains that are ligands for langerin detected on the glycan array, were solved. Because several of the structures show the same interactions, to simplify presentation of the results, only the data sets that gave the highest resolution structures are shown: wild type langerin CRD bound to α-Me-GlcNAc and trisaccharide TR54, langerin-I313 CRD bound to α-Me-GlcNAc and trisaccharide TR54, and langerin-D288/I313 CRD bound to α-Me-GlcNAc, α-Me-Man and trisaccharide TR55 (Tables 3 and 4).

**TABLE 3:** **Crystallographic data statistics**

	Langerin CRD + Me-GlcNAc	Langerin CRD + TR54	Langerin CRD-I313 + Me-GlcNAc	Langerin CRD-I313 + TR54	Langerin CRD-D288/I313 + Me-GlcNAc	Langerin CRD-D288/I313 + Me-Man	Langerin CRD-D288/I313 + TR55
Wavelength	0.97650	0.97945	0.97650	0.97945	0.97945	0.97945	0.97945
Unit cell lengths (Å)	*a* = *b* = 79.89, *c* = 90.47	*a* = *b* = 79.42, *c* = 91.71	*a* = *b* = 80.06, *c* = 90.17	*a* = *b* = 79.49, *c* = 91.93	*a* = *b* = 79.55, *c* = 89.88	*a* = *b* = 79.92, *c* = 90.36	*a* = *b* = 79.03, *c* = 91.39
Resolution Å (last shell)	47.92-1.75 (1.84-1.75)	36.44-1.85 (1.95-1.85)	47.94-1.75 (1.84-1.75)	36.48-1.85 (1.95-1.85)	39.13-1.85 (1.95-1.85)	39.33-2.00 (2.11-2.00)	39.56-2.00 (2.11-2.0)
*R*_sym_ (last shell)*^a^*	5.5 (22.8)	4.0 (18.8)	3.5 (8.8)	3.7 (16.6)	3.6 (23.2)	5.8 (31.7)	3.9 (21.8)
Mean((*I*)/σ(*I*)) (last shell)	21.8 (7.4)	27.8 (9.2)	37.5 (17.2)	30.8 (10.5)	23.2 (5.6)	19.1 (4.9)	25.1 (6.6)
% of completeness (last shell)	99.4 (98.8)	97.6 (97.5)	98.7 (97.9)	98.8 (99.4)	99.2 (98.5)	99.4 (98.4)	99.1 (98.7)
Number of unique reflections	56,914	47,282	56,552	48,723	47,327	38,155	37,595
Average multiplicity (last shell)	7.2 (7.1)	6.9 (7.0)	7.4 (7.2)	6.7 (6.8)	3.8 (3.8)	4.5 (4.4)	4.6 (4.6)

*^a^ R*_sym_ = Σ*_h_*Σ*_i_* (|*I_i_*(*h*)| − |〈*I*(*h*)〉|)/Σ*_h_*Σ*_i_ I_i_*(*h*) where *I_i_*(*h*) = observed intensity, and 〈*I*(*h*)〉 = mean intensity obtained from multiple measurements.

**TABLE 4:** **Crystallographic refinement statistics**

	Langerin CRD + Me-GlcNAc	Langerin CRD + TR54	Langerin CRD-I313 + Me-GlcNAc	Langerin CRD-I313 + TR54	Langerin CRD-D288/I131 + Me-GlcNAc	Langerin CRD-D288/I131 + Me-Man	Langerin CRD-D288/I131 + TR55
Number of reflections used for refinement	53,793	44,431	53,381	45,546	44,894	36,132	35,498
Reflections marked for *R*_free_	2887	2366	2858	2438	2407	1908	1876
*R*_free_*^a^*	23.3	21.8	22.8	21.2	21.4	24.6	23.3
*R*_cryst_*^a^*	18.0	17.3	17.6	16.6	17.0	18.9	18.1
Average B factor (Å^2^)	25.2	23.3	22.2	22.5	21.7	23.0	28.2
Bond length r.m.s.d.*^b^* (Å)	0.007	0.008	0.007	0.007	0.007	0.006	0.008
Angle r.m.s.d.*^b^* (°)	1.1	1.1	1.1	1.1	1.1	0.94	1.1
Ramachandran plot: (% in each region)*^c^*							
Preferred	96.0	96.2	95.8	95.8	95.4	96.0	95.0
Allowed	4.0	3.8	4.2	4.2	4.6	3.8	5.0
Outliers	0	0	0	0	0.0	0.2	0.0

*^a^ R* and *R*_free_ = Σ ‖*F_o_*| − |*F_c_*‖/Σ|*F_o_*|, where |*F_o_*| = observed structure factor amplitude and |*F_c_*| = calculated structure factor amplitude for the working and test sets, respectively.

*^b^* r.m.s.d., root mean square deviation.

*^c^* As defined in Coot.

**FIGURE 5.**
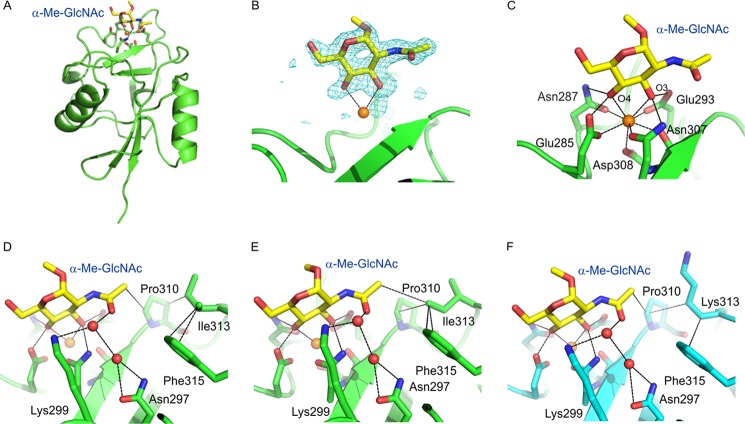
**Structures of langerin CRDs bound to α-Me-GlcNAc.** *A*, overall view of α-Me-GlcNAc bound to the CRD portion of Langerin-I313. *B*, *F_o_* − *F_c_* electron density for the αMe-GlcNAc bound to Langerin-I313, calculated by omitting the sugar residue from the model, contoured at 3.5 σ. *C*, interactions of α-Me-GlcNAc at the principal Ca^2+^ site of Langerin-I313. *D*, interactions of the *N*-acetyl substituent of α-Me-GlcNAc bound to Langerin-I313. *E*, interactions of the *N*-acetyl substituent with an alternative rotamer of Ile-313. This rotamer is seen in one of four monomers in the crystals of langerin-I313 complexed with α-Me-GlcNAc and in two of four monomers in the crystals of langerin-I313 complexed with trisaccharide TR54 and langerin-D288/I313 complexed with trisaccharide TR55. *F*, an equivalent view of αMe-GlcNAc bound to the CRD from wild type langerin. Ca^2+^ is shown in *orange*, oxygen atoms are *red*, and nitrogen atoms are *blue*. Hydrogen bonds and van der Waals contacts were assigned with maximum distance cutoffs of 3.4 and 4.2 Å, respectively. Hydrogen bonds, coordination bonds, and van der Waals contacts are indicated by *dotted lines*.

The position of α-Me-GlcNAc bound to the CRD of langerin-I313 (Fig. 5, *A–C*), is analogous the position of mannose in the wild type CRD described previously (18). The 3- and 4-OH groups of the sugar are coordinated to the principal Ca^2+^ and form hydrogen bonds with Glu-293 and Asn-307 and with Asn-287 and Glu-285, respectively. However, the equatorial *N*-acetyl substituent at the two position of GlcNAc makes several additional secondary contacts, including water-mediated hydrogen bonds with Lys-299 and Asn-297. In addition, there are van der Waals contacts with Pro-310, which in turn interacts with Ile-313 (Fig. 5*D*). An alternate rotamer of the Ile-313 side chain brings it into direct van der Waals contact with the *N*-acetyl group of the GlcNAc (Fig. 5*E*). A similar set of contacts is seen in the structure of α-Me-GlcNAc bound to the wild type CRD, with the side chain of Lys-313 taking the place of the isoleucine side chain (Fig. 5*F*). However, the Lys-313 side chain has weak electron density, suggesting flexibility. Modeling shows that some Lys-313 rotamers would occlude the sugar-binding site, and in the absence of the intervening sulfate moiety that is present in 6SO_4_-Gal, there might be electrostatic repulsion between Lys-313 and Lys-299. One or more of these factors may explain the increased binding of the Ile-313 variant to GlcNAc.

**FIGURE 6.**
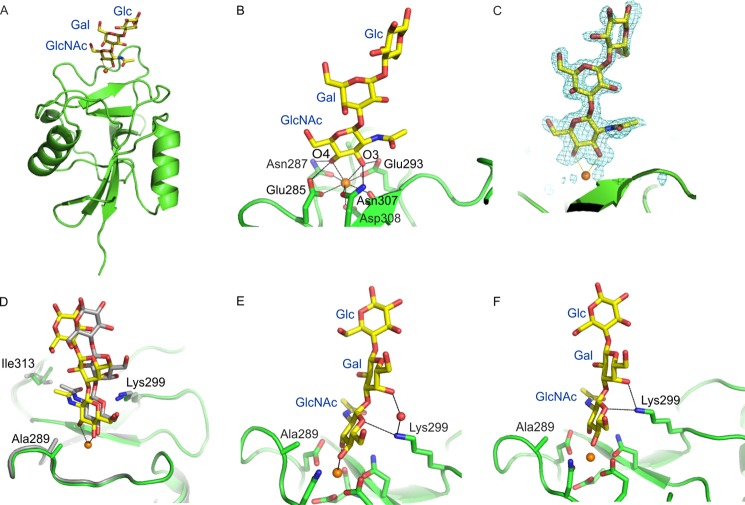
**Structures of the CRD of langerin-I313 bound to GlcNAc-β1-3Galβ1-4Glc.** *A*, overall view of the oligosaccharide-CRD complex. *B*, expanded view of the oligosaccharide interactions at the principal Ca^2+^ site. *C*, *F_o_* − *F_c_* electron density for the bound oligosaccharide, calculated by omitting the sugar residues from the model, contoured at 3.0 σ. *D*, superposition of the bound oligosaccharide in copy A (*yellow*) and D (*gray*). *E*, view of oligosaccharide bound to monomer A, showing interactions of the protein with the galactose residue. *F*, view of oligosaccharide bound to copy D. Ca^2+^ is shown in *orange*, oxygen atoms are *red*, and nitrogen atoms are *blue*.

In the structures of the Ile-313 variant bound to a GlcNAc-terminating trisaccharide, most of the glycan projects away from the surface of the CRD (Fig. 6, *A* and *B*). Although the position of the oligosaccharide is well defined in the electron density map (Fig. 6*C*), there are differences in the position of the oligosaccharide in the different copies in the crystallographic asymmetric unit (Fig. 6*D*), probably resulting from crystal contacts. This observation suggests that there may be alternative orientations of the residues beyond the GlcNAc. However, in all the observed orientations, there are hydrogen bonds between the subterminal galactose residue and the side chain of Lys-299 (Fig. 6, *E* and *F*). These contacts would not be affected by the variations at positions 288 or 313.

Structures of the double mutant, with Asp-288 as well as Ile-313, with α-Me-GlcNAc, and with the extended GlcNAc-containing oligosaccharides, showed exactly the same pattern of interactions of the *N*-acetyl group as were observed for the Ile-313 mutation alone. Thus, the differences in the interactions of Lys-313 and Ile-313 described for the single site mutant can account for the enhanced binding of GlcNAc to the naturally occurring Asp-288/Ile-313 variant as compared with the wild type CRD.

**FIGURE 7.**
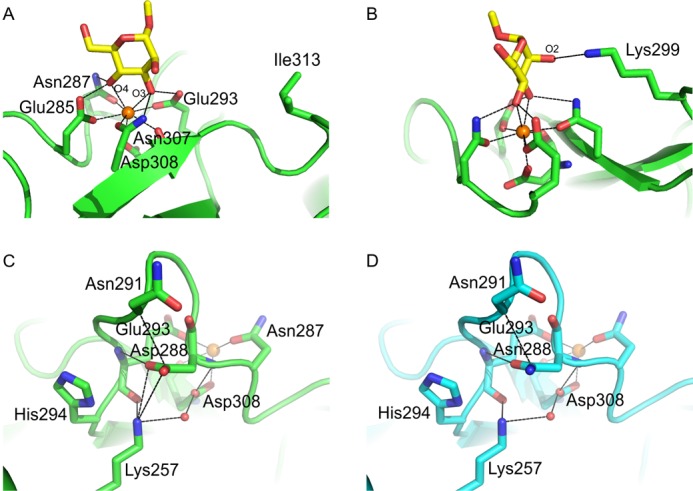
**Structure of the CRD of langerin-D288/I313.** *A*, structure of langerin-D288/I313 CRD complexed with α-Me-Man, showing the interactions of the 3- and 4-OH groups with the principal Ca^2+^. *B*, same structure as in *A*, rotated to show additional interactions with the 2-OH group of mannose. *C* and *D*, langerin-D288/I313 CRD (*C*) and wild type langerin CRD (*D*) complexed with α-Me-GlcNAc, showing changes in the hydrogen bonds associated with residue 288 as a result of the Asn/Asp polymorphism. Ca^2+^ is shown in *orange*, oxygen atoms are *red*, and nitrogen atoms are *blue*.

To investigate the structural basis for the reduced binding of mannose-containing structures to the Asp-288/Ile-313 variant, the structure of this form of the CRD in complex with α-Me-Man was determined (Fig. 7, *A* and *B*). The interactions of mannose with the principal Ca^2+^ through the 3- and 4-OH groups, as well as the additional interaction of the 2-OH group with the side chain of Lys-299, are the same as those observed for the wild type CRD (18). The Ile-313 side chain is distant from the mannose, consistent with the conclusion that this residue enhances binding to GlcNAc but plays little role in the affinity for mannose because the Asp-288 mutant alone shows a similar loss of affinity. Residue 288 is much closer to the principal Ca^2+^ site (Fig. 7, *C* and *D*). The position of the side chain of Lys-257 remains the same in the presence of either aspartic acid or asparagine at position 288, and it does not have appropriate geometry to form hydrogen bonds with the side chain at position 288. However, the electrostatic interactions would be significantly different with aspartic acid at position 288 because this side chain forms a salt bridge with the amino group of Lys-257, which in turn forms part of a water-mediated hydrogen bond network with the side chain of Asp-308 (Fig. 7*C*). Interactions with both backbone and side chain oxygen atoms of Asp-308 form a key part of the Ca^2+^-binding site. Thus, subtle differences in the hydrogen-bonding pattern resulting from the change of asparagine to aspartic acid at this position, could account for the reduced affinity for Ca^2+^, which in turn leads to an overall reduction in affinity for sugars (22).

## DISCUSSION

The N288D and K313I polymorphisms in langerin provide the first example of naturally occurring amino acid changes that alter the glycan specificity of a glycan-binding receptor. Overall, the combination of these two amino acid changes switches the specificity of langerin from showing approximately equal binding to high mannose structures, fucosylated blood group antigens, and glycans with terminal 6SO_4_-Gal, to binding mainly to glycans with terminal GlcNAc. The frequency of the allele containing Asp-288 and Ile-313 is quite high; overall 1% of individuals are homozygotes, and this value is higher in some African populations. Individuals homozygous for the Asp-288/Ile-313 allele would produce langerin molecules that could not bind glycans with terminal 6SO_4_-Gal. Because langerin functions as a homotrimer, heterozygotes for the Asp-288/Ile-313 allele as well as homozygotes would be expected to have langerin with altered carbohydrate binding specificity. Assuming equal synthesis of polypeptides from the two chromosomes and random association of the polypeptides, in heterozygotes, only one in eight langerin molecules would have three CRDs containing Asn-288 and Lys-313 and one in eight langerin molecules would have three CRDs containing Asp-288 and Ile-313. The remaining langerin trimers in heterozygotes would have two CRDs with Asn-288 and Lys-313 and one with Asp-288 and Ile-313 (three-eighths of the total langerin molecules) or one CRD with Asn-288 and Lys-313 and two with Asp-288 and Ile-313 (three-eighths of the total langerin molecules). Thus, heterozygotes would potentially have langerin oligomers with a range of differing carbohydrate specificities. Although the effects of this range of specificities remain to be investigated, having multiple types of binding sites might provide an advantage in defense against a broad range of pathogens.

The physiological function of 6SO_4_-Gal binding by langerin is not yet clear, although keratan sulfate is a potential endogenous ligand containing 6SO_4_-Gal. Binding of langerin to keratan sulfate from shark cartilage has been demonstrated, and roles of langerin binding to keratan sulfate in cell adhesion or tumor surveillance have been proposed (9). In the linear repeating Galβ1-4GlcNAcβ1-3 structure of keratan sulfate, multiple galactose residues as well as GlcNAc residues can be sulfated at the 6-position (25). However, because binding of 6SO_4_-Gal to the langerin CRD requires ligation of the 3- and 4-OH groups, only 6SO_4_-Gal at the termini of keratan sulfate chains could bind. The 3-OH groups of internal 6SO_4_-Gal residues are linked to GlcNAc, preventing binding to langerin. The nonreducing ends of keratan sulfate chains are often capped with α2-3-linked sialic acid, which would also prevent binding to langerin. Langerin was shown to bind to Chinese hamster ovary cells expressing large amounts of keratan sulfate following transfection with both keratan sulfate 6-*O* sulfotransferase (KS6GalST) and GlcNAc 6-*O* sulfotransferase (GlcNAc6ST), the two enzymes that transfer sulfate to the 6-position of galactose and GlcNAc, respectively (9). Binding was much greater if the cells were first treated with a sialidase, consistent with the presence of sialic acid capping groups preventing binding to keratan sulfate. Langerin only binds to a glioblastoma cell line expressing large amounts of keratan sulfate following sialidase treatment, again emphasizing the fact that terminal 6SO_4_-Gal residues on keratan are not common (9). In the same study, langerin bound to mouse spleen cells without sialidase treatment. Such binding could be due to the presence of 6SO_4_-Gal-containing ligands because spleen expresses high levels of keratan sulfate 6-*O*-sulfotransferase as compared with other tissues, but high mannose or fucosylated glycans for langerin could also have been present on the cells. Core 1 and Core 2 *O*-linked glycans of mucins and mucin-like glycoproteins occasionally have terminal 6SO_4_-Gal residues that would also be potential endogenous ligands for langerin, although again most commonly 6SO_4_-Gal residues would be capped with α2-3-linked sialic acid, preventing binding to langerin (26, 27).

It is possible that under certain pathological conditions, undersialylation of glycans would leave more terminal 6SO_4_-Gal exposed for recognition by langerin. There could also be unique 6SO_4_-Gal-terminated glycans on specific proteins that have not yet been identified. In the absence of a clear role for 6SO_4_-Gal recognition by langerin, it is difficult to predict the effect that complete loss of this function might have. However, what is currently known suggests that the 6SO_4_-Gal binding activity of langerin could be dispensable, and individuals homozygous for the Asp-288/Ile-313 form of langerin may not be disadvantaged by loss of this activity.

The function of recognition of nonsulfated carbohydrate ligands by langerin is clearer because these structures are found on the surfaces of pathogens, and binding and uptake of several microorganisms by langerin has been demonstrated. Langerin binds viruses such as HIV and herpes simplex 2 through high mannose structures present on envelope glycoproteins and can also bind α1-2-linked mannose residues present in the mannans of fungal cell walls and on bacterial lipopolysaccharides (5, 6, 8, 9, 18). The fucosylated blood group B and A trisaccharides recognized by langerin are also found on pathogenic bacteria, such as *Helicobacter pylori* and *Escherichia coli* (28). GlcNAc residues are also common on the surfaces of microorganisms, on the lipopolysaccharides of Gram-negative bacteria and the teichoic acids characteristic of Gram-positive bacteria, as well as in chitin in the cell walls of fungi (29).

The linkage of the two polymorphisms in langerin may provide insight into co-evolution with pathogens. The D288N change affects the stability of the primary binding site and reduces the affinity of the CRD for monosaccharides. If this mutation arose first, the change of Lys-313 to Ile may have arisen subsequently in langerin with Asp-288 so that the packing interaction of the Ile side chain with the *N*-acetyl group of GlcNAc could compensate for reduced affinity at the Ca^2+^ site and allow recognition of pathogenic bacteria. Prevalence of pathogenic bacterial strains with more GlcNAc in their cell wall lipopolysaccharides or teichoic acids may then have selected for the Asp-288/Ile-313 langerin allele in some populations.

The enhanced affinity for GlcNAc caused by the K313I change may be advantageous in allowing more efficient uptake of certain pathogens. Heterozygotes for the Asp-288/Ile-313 allele would have some langerin molecules with enhanced affinity for GlcNAc while retaining some langerin molecules with higher affinity for high mannose structures and fucosylated glycans as well as 6SO_4_-Gal binding activity, potentially allowing protection against a wider range of microorganisms. In contrast to some other C-type lectins involved in pathogen recognition such as DC-SIGN, which show considerable structural and functional divergence between species (30), langerin is well conserved in mammalian species. The existence of common natural variants within a species may represent an alternative way of adapting to the challenge of different pathogens.

## Footnotes

2 The abbreviations used are:

CRD: carbohydrate recognition domain
6SO_4_-Gal: 6-sulfated galactose.
